# Temporal trends in molecular markers of drug resistance in *Plasmodium falciparum* in human blood and profiles of corresponding resistant markers in mosquito oocysts in Asembo, western Kenya

**DOI:** 10.1186/s12936-022-04284-6

**Published:** 2022-09-13

**Authors:** Zhiyong Zhou, John E. Gimnig, Sheila B. Sergent, Ying Liu, Bernard Abong’o, Kephas Otieno, Winnie Chebore, Monica P. Shah, John Williamson, Feiko O. ter Kuile, Mary J. Hamel, Simon Kariuki, Meghna Desai, Aaron M. Samuels, Edward D. Walker, Ya Ping Shi

**Affiliations:** 1 Division of Parasitic Diseases and Malaria, Center for Global Health, Centers for Disease Control and Prevention, Atlanta, GA USA; 2 Centre for Global Health Research, Kenya Medical Research Institute, Kisumu, Kenya; 3 Department of Clinical Sciences, Liverpool School of Tropical Medicine, Liverpool, UK; 4 Division of Parasitic Diseases and Malaria, Center for Global Health, Centers for Disease Control and Prevention, Kisumu, Kenya; 5 Department of Microbiology and Molecular Genetics, Michigan State University, East Lansing, MI USA

**Keywords:** *Plasmodium falciparum*, Resistant markers, Sextuple *dhfr/dhps* mutant, *Pfcrt*, *Pfmdr1*, *Pfk13*, Trend, Mosquitoes, Asembo, Western Kenya

## Abstract

**Background:**

Over the last two decades, the scale-up of vector control and changes in the first-line anti-malarial, from chloroquine (CQ) to sulfadoxine-pyrimethamine (SP) and then to artemether-lumefantrine (AL), have resulted in significant decreases in malaria burden in western Kenya. This study evaluated the long-term effects of control interventions on molecular markers of *Plasmodium falciparum* drug resistance using parasites obtained from humans and mosquitoes at discrete time points.

**Methods:**

Dried blood spot samples collected in 2012 and 2017 community surveys in Asembo, Kenya were genotyped by Sanger sequencing for markers associated with resistance to SP (*Pfdhfr, Pfdhps)*, CQ, AQ, lumefantrine (*Pfcrt, Pfmdr1)* and artemisinin (*Pfk13).* Temporal trends in the prevalence of these markers, including data from 2012 to 2017 as well as published data from 1996, 2001, 2007 from same area, were analysed. The same markers from mosquito oocysts collected in 2012 were compared with results from human blood samples.

**Results:**

The prevalence of SP *dhfr/dhps* quintuple mutant haplotype C_50_**I**_51_**R**_59_**N**_108_I_164_/S_436_**G**_437_**E**_540_A_581_A_613_ increased from 19.7% in 1996 to 86.0% in 2012, while an increase in the sextuple mutant haplotype C_50_**I**_51_**R**_59_**N**_108_I_164_/**H**_436_**G**_437_**E**_540_A_581_A_613_ containing *Pfdhps*-436H was found from 10.5% in 2012 to 34.6% in 2017. Resistant *Pfcrt*-76 T declined from 94.6% in 2007 to 18.3% in 2012 and 0.9% in 2017. Mutant *Pfmdr1*-86Y decreased across years from 74.8% in 1996 to zero in 2017, mutant *Pfmdr1*-184F and wild *Pfmdr1*-D1246 increased from 17.9% to 58.9% in 2007 to 55.9% and 90.1% in 2017, respectively. *Pfmdr1* haplotype N_86_**F**_184_S_1034_N_1042_D_1246_ increased from 11.0% in 2007 to 49.6% in 2017. No resistant mutations in *Pfk13* were found. Prevalence of *Pfdhps*-436H was lower while prevalence of *Pfcrt-*76 T was higher in mosquitoes than in human blood samples.

**Conclusion:**

This study showed an increased prevalence of *dhfr/dhps* resistant markers over 20 years with the emergence *of Pfdhps*-436H mutant a decade ago in Asembo. The reversal of *Pfcrt* from CQ-resistant to CQ-sensitive genotype occurred following 19 years of CQ withdrawal. No *Pfk13* markers associated with artemisinin resistance were detected, but the increased haplotype of *Pfmdr1* N_86_**F**_184_S_1034_N_1042_D_1246_ was observed. The differences in prevalence of *Pfdhps*-436H and *Pfcrt-*76 T SNPs between two hosts and the role of mosquitoes in the transmission of drug resistant parasites require further investigation.

**Supplementary Information:**

The online version contains supplementary material available at 10.1186/s12936-022-04284-6.

## Background

Over the last two decades, increased malaria control efforts have saved millions of lives globally and reduced malaria mortality in Africa by 44.0% [1]. Like other malaria endemic areas, malaria control has significantly decreased malaria morbidity and all-cause mortality in western Kenya [1, 2] by increasing access to effective anti-malarial treatment and scale-up of long-lasting insecticide-treated nets (LLINs) [2]. However, progress is threatened by the potential emergence and spread of anti-malarial drug resistance.

In Kenya, Ministry of Health guidelines for the treatment of malaria have changed in response to the spread of drug resistant parasites. Due to widespread CQ resistance, sulfadoxine-pyrimethamine (SP) replaced chloroquine (CQ) as first-line treatment for uncomplicated malaria in 1998. However, resistance to SP rapidly developed and in 2004, artemether-lumefantrine (AL), an artemisinin-based combination therapy (ACT), was recommended as the first-line anti-malarial for uncomplicated malaria [3]. Because of AL supply shortage, amodiaquine (AQ) was temporarily used as first-line treatment between 2004 and 2006 [4]. SP remains the drug of choice for intermittent preventive treatment in pregnancy (IPTp), which has been widely implemented in western Kenya [5].

In Asembo, Siaya County, western Kenya, malaria vector control started in 1997 with a large-scale trial of insecticide-treated nets (ITNs). Nationally, ITNs were initially distributed to pregnant women and children through social marketing schemes. In 2006, the first mass distribution campaign of long-lasting insecticidal nets (LLINs) was implemented to target children under 5 years of age and pregnant women. Subsequent mass campaigns in 2011 and 2014 targeted all age groups to achieve universal coverage. The scale-up of ITNs was associated with changes in the predominant mosquito species with substantial reductions in *Anopheles gambiae *sensu stricto (s.s.) and *Anopheles funestus* that resulted in a predominance of *Anopheles arabiensis* [6]. However, *An. funestus* has resurged and became the predominant species since 2010 in the Asembo area [7]. It is unknown whether the temporal changes in mosquito vector species influenced the spread of drug resistant parasites in mosquito populations in addition to anti-malarial drug pressures in humans in this area.

Molecular surveillance is essential to detect trends in known drug-resistant mutations as well as emerging mutations. Single nucleotide polymorphisms (SNPs) in the genes for dihydrofolate reductase (DHFR) and dihydropteroate synthase (DHPS) are known to cause resistance to SP. Triple mutations of *Pfdhfr* 51I, 59R, and 108 N combined with double mutations of *Pfdhps* 437G and 540E (a quintuple set of mutations) are strongly associated with SP treatment failure [8], and a high prevalence of sextuple-mutant parasites containing the 581G mutation (*Pfdhfr* I_51_R_59_N_108_ and *Pfdhps* G_437_E_540_G_581_) has been associated with reduced IPTp-SP efficacy in sub-Saharan Africa [9]. *Plasmodium falciparum* chloroquine resistance transporter gene (*Pfcrt*) 76 T and multi-drug resistant gene 1 (*Pfmdr1*) 86Y are key molecular markers associated with the emergence and spread of resistance to CQ and AQ [10, 11]. In the Greater Mekong Subregion, multiple SNP mutations in the *Kelch 13* gene *(Pfk13)* are associated with resistance to artemisinin, the drug currently used widely in ACTs [12–14]. In Africa, the selection of wild type *Pfmdr1-*N86 and the haplotype N_86_F_184_D_1246_ by ACT partner drug, lumefantrine, has been documented [15, 16] with a possible association with reduced susceptibility to treatment [15, 17, 18]. More recent reports show that artemisinin resistant mutation *Pfk13* R561H in Rwanda and *Pfk13* A675V and C469Y in Uganda are associated with delayed clearance of malaria parasites [19, 20].

The spread of resistant parasites also depends on non-drug pressure factors, including immune clearance in human host, multiplicity of infection, fitness cost in both human and vector, vector species, genetic recombination, and mid-gut microbiota in mosquitoes [21–23]. Previous studies conducted in different transmission settings have suggested that selection of some drug-resistant molecular markers in malaria parasites may occur during transmission by mosquitoes [24–26]. Different drug-resistant mutations of parasites selected in humans and mosquitoes have been reported in some studies [25, 26] while similar molecular resistant profiles of *Pfdhfr, Pfdhps, Pfk13, Pfcrt, Pfmdr1* between human and mosquito populations were found in others [27, 28]. The different results from different malaria endemic areas warranted the examination of whether profiles of molecular markers of parasite drug resistance in mosquitoes differ from those in human blood in this high transmission area of western Kenya.

To evaluate the impact of malaria control on drug-resistant markers in the Asembo area of western Kenya, samples from community cross-sectional surveys in 1996, 2001, and 2007 were previously tested for molecular markers of resistance [29, 30]. Those studies showed that the prevalence of molecular markers of resistance to CQ and SP continued to increase even after withdrawal of these drugs as first-line treatment and with the sustained use of ITNs in western Kenya [30]. The present study extends these observations to include survey samples from 2012 to 2017 by testing the resistance markers associated with previously and currently used anti-malarial drugs to further evaluate the temporal trends in these drug-resistance markers between 1996 and 2017. This study also compares the prevalence of molecular markers of drug resistance between *P. falciparum* parasites isolated from mosquito oocysts and from human blood samples collected in 2012.

## Methods

### Ethics statement

This study was approved by the Ethical Review Committee of KEMRI, Nairobi, Kenya, the Institutional Review Board of Michigan State University, East Lansing, Michigan, and the Institutional Review Board of the Centers for Disease Control and Prevention (CDC), Atlanta, USA.

### Study area and sample collection

This study was part of a series of community-based household surveys to evaluate the impact of malaria control interventions within the Kenya Medical Research Institute and Centers for Disease Control and Prevention (KEMRI/CDC) Health and Demographic Surveillance System (HDSS) in Asembo, Siaya County, western Kenya [31]. Surveys carried out in 2012 and 2017 utilized systematic random selection of households to estimate population representative prevalence. Among households selected, all individuals above 1 months of age were eligible for inclusion. Dried blood spot (DBS) from smear-positive participants between June and August from the survey in 2012 (N = 225) and between June and September in the 2017 survey (N = 121) were used for this parasite genotyping study.

Mosquitoes were collected using prokopack aspiration of indoor resting mosquitoes [32] from May to September in 2012 in a single village in the Asembo area. Approximately 20 houses were sampled each morning from 7 to 11 am. Collections were done 5 days during one week each month with collectors using prokopack aspirators to capture mosquitoes resting on the walls, the roof, under furniture and from hangings within the house. Collectors spent 15–20 min in each house before moving to a new house. Houses were selected non-randomly with the aim to maximize mosquito collections but efforts were made to ensure that all houses within the village were sampled at least once during the collection period.

Collected mosquitoes were transferred into adult mosquito cage (30 × 30× 30 cm) from where the fed, gravid and half gravid female anophelines were sorted out and held separately in paper cups while the rest were killed and discarded in the field. Fed, gravid and half gravid female mosquitoes collected from the field were returned to the KEMRI/CDC facilities in Kisian and held in the insectary, sustained with sugar solution. Blood fed and half-gravid mosquitoes were held for 6 to 8 daysbefore oocyst dissection, while gravid females were dissected after 4 days. The identified oocysts were preserved in absolute ethanol and stored at − 20 °C, then shipped to the CDC malaria lab in Atlanta for sequencing. Because the midgut was preserved in absolute ethanol, the midgut sample was dried on filter paper and transferred to a 1.5 ml tube and ground with a sterile blue Kontes pestle in lysis buffer before DNA extraction. Before dissection, mosquitoes were identified morphologically using the published keys for the Anophelinae of Africa [33]. Mosquitoes from the *An. gambiae* complex were further identified as *An. gambiae *s.s*.* or *Anopheles arabiensis* using the methods of Scott et al. [34].

### DNA extraction

A whole spot (50 µl blood) from a DBS sample was cut from which DNA was extracted. Parasite genomic DNA from DBS samples was extracted using QIAamp^®^ DNA Mini Kit (QIAGEN, Valencia, CA) and from the mosquito midguts using QIAmp DNA Micro kit following manufacturer’s instructions (QIAGEN). Extracted DNA samples were stored at − 20 °C until use.

### Nested PCR amplification for *Pfdhfr* and *Pfdhps* genes

Nested PCR was used to amplify the *Pfdhfr* gene covering codons 51, 59, 108, and 164 and the *Pfdhps* gene covering codons 436, 437, 540, 581 and 613 for all blood and mosquito samples collected. Primers were designed using Primer3web (Github Inc, San Francisco, CA) and synthesized at the CDC Core Facility in Atlanta, GA. All primary and secondary PCRs were performed in a 25 µl reaction with Premixed 2 × PCR Master Mix (Promega Corporation, Madison, WI). For both of *Pfdhfr* and *Pfdhps* primary PCR, a 25 µl reaction contained 12.5 µl 2 × master mix, 1 µl (400 nM) each primer, 2 µl DNA template and 8.5 µl PCR-grade water. For secondary PCR, reaction system is the same except for template with 2 µl primary PCR products for both genes. After secondary PCRs, 1% agarose gel was prepared and gel electrophoreses were run for the amplification of sample PCR bands, and positive, negative and blank controls. The final PCR products of 542 bp and 675 bp of *Pfdhfr* and *Pfdhps* genes, respectively, were used for Sanger sequencing. The PCR primers and recycling conditions are shown in Additional file 1: Table S1.

### Nested PCR amplification for *Pfcrt*, *Pfmdr1* and *Pfk13* genes

The primers and PCR conditions used for *Pfcrt, Pfmdr1* and *Pfk13* amplification were adapted from previously published work [35–37] (see Additional file 2: Table S2). For *Pfmdr1* gene, the first fragment, covering codons 86 and184, was amplified using the same primers as shown in the publication [36]. The second fragment amplification covering codons 1034, 1042 and 1246 was modified by adding 2 internal primers (forward primer: 5ʹGCAATCGTTGGAGAAACAGG-3ʹ and reverse primer: 5ʹTTTTGCATTTTCTGAATCTCCTT-3ʹ) in the secondary PCR. Thus, the final secondary PCR products included a 298 bp amplicon covering codons 1034–1042 and a 414 bp amplicon covering codon 1246 were produced in the current study. After secondary PCR, 1% agarose gel was prepared and gel electrophoreses were run for the amplification of sample PCR bands, and positive, negative and blank controls.

### Sanger sequencing of amplified drug-resistant genes

Nested PCR products were purified with ExoSAP (New England Biolabs, Ipswich, MA, USA). The cycling sequencing was carried out using Big Dye Terminator v3.1 cycle sequencing kit on Bio-Rad C1000 thermal cycler (Bio-Rad, Hercules, CA, USA). Dye terminators were cleaned by precipitating reactions with EDTA/NaOAc/Ethanol precipitation and rehydrating in 10 μl HiDi formamide. Capillary electrophoresis was performed in ABI 3730 or 3730xl genetic analyzer (Applied Biosystems, Foster City, USA). Sequence data were analysed using the Geneious Prime R10 and R11 (Biomatters, San Francisco, CA, USA). The mixed SNPs were identified where the minor peak was ≥ 30% of the major peak. SNPs at *Pfdhfr, Pfdhps, Pfcrt, Pfmdr1 genes and Pfk13* propeller region were identified by comparing sequences with reference 3D7 strains from GenBank XM_001351443.1, Z30654.1, XM_001348968.1, XM_001351751 and MW712622.1), respectively. PCR reaction systems and cycling conditions were detailed in Additional files 1, 2.

### Genetic definitions

As with previous studies [29, 30], all SNPs related to drug resistance molecular markers were classified as either wild, mutant or mixed. When calculating the prevalence and constructing haplotypes, mixed genotypes were considered as resistant mutant [30]. The *Pfdhfr* haplotypes in codons 50, 51, 59, 108 and 164 were classified as wild type, single, double, triple, and quadruple mutants. The *Pfdhps* haplotypes in codons 436, 437, 540, 581 and 613 were classified as wild type, single, double and triple haplotypes. The combined *dhfr/dhps* haplotypes (codons of *Pfdhfr* 50, 51, 59, 108, 164 and *Pfdhps* 436, 437, 540, 581, 613) were classified as wild type, single, double, triple, quadruple, quintuple and sextuple mutants. *Pfcrt* haplotypes based on codons 72, 73, 74, 75, 76 and *Pfmdr1* haplotypes based on codons 86, 184, 1034, 1042, 1246 were also constructed. The SNPs in *Pfk13* domain at nine codons 446, 458, 476, 493, 539, 543, 553, 561, 580 were assessed for the presence of mutations known to be associated with artemisinin resistance by the World Health Organization (WHO) 2018 standards [38].

### Statistical analysis

Because age is not a confounding factor for the prevalence of drug-resistant molecular markers in our previous study [30], analyses of temporal trends between different sampling years were not adjusted for age in this study. The temporal trends in the prevalence of resistant markers were analysed using the exact version of the Cochran-Armitage test for trend. Differences in the prevalence of SNPs and haplotypes between mosquito and human samples in 2012 survey were assessed using Pearson Chi-square tests (exact version) and exact 95% confidence intervals (CIs) for the prevalence of SNPs and haplotypes in mosquitoes and human samples were also calculated. These statistical analyses were conducted using SAS v9.4. All statistical tests were two-tailed and statistical significance was defined as p < 0.05. Marginal differences were defined as 0.05 ≤ p < 0.10. The prevalence of SNPs and haplotypes were not adjusted for sampling weights.

## Results

### Prevalence of *Pfdhfr* and *Pfdhps* resistant SNPs and haplotypes in 2012 and 2017 surveys

The prevalence of SP resistance mutations at *Pfdhfr*-51I, -59R, -108 N and *Pfdhps*-437G, -540E approached fixation (96.4–100%) with no significant differences in frequencies observed between 2012 and 2017 (Table 1), while the prevalence of the *Pfdhps*-436H mutation increased significantly from 10.5% in 2012 to 34.6% in 2017 (p = 0.001). In contrast, the *Pfdhfr*-164L mutation which was detected previously in a cotrimoxazole prophylaxis study in the Kisumu region of western Kenya [39] and the *Pfdhps*-581G mutation which was reported in patients registered at the outpatient clinic of New Nyanza Provincial Hospital, Kisumu, Kenya [40], were observed in only two samples in the 2012 survey (0.9% and 1.0%, respectively) and none in 2017 in the Asembo area (Table 1). There was no *Pfdhfr-*164L mutation found in Asembo in 1996, 2001, or 2007 [29, 30] and no *Pfdhps-*581G mutation found in 2007 [30] (codon *Pfdhps-*581 was not tested in the 1996 & 2001 surveys). Comparison of mutant haplotypes between 2012 and 2017 showed no significant increase in the prevalence of all haplotypes but a significant increase in *Pfdhps* triple mutants (**H**_**436**_**G**_**437**_**E**_**54**0_A_581_A_613_), reflecting the increase in the *Pfdhps*-436H mutation (p = 0.001). In parallel, the prevalence of the combined haplotype of *dhfr/dhps* sextuple mutant C_50_**I**_51_**R**_59_**N**_108_I_164_/**H**_436_**G**_437_**E**_540_A_581_A_613_ containing *Pfdhps*-436H was 10.5% in 2012 and 34.6% in 2017 (p = 0.001, Table 1).

**Table 1:** Prevalence of SP mutant SNPs and haplotypes between 2012 and 2017

Gene	SNP	Percent in 2012	Percent in 2017	P value
*Pfdhfr*		n = 212	n = 111	
	C50R	0	0	N/A
	N51I	100	100	1.000
	C59R	97.2	96.4	0.965
	S108N	100	100	1.000
	I164L	0.9	0	0.779
*Pfdhps*		n = 210	n = 111	
	S436H	10.5	34.6	**0.001**
	A437G	100	100	1.000
	K540E	99.5	97.2	0.221
	A581G	1.0	0	0.792
	A613T	0	0	N/A

Mutation number	Haplotype	Percent in 2012	Percent in 2017	P value
*Pfdhfr*		n = 212	n = 111	
Wild	C_50_N_51_C_59_S_108_I_164_	0	0	N/A
Double	C_**50**_**I**_51_C_59_**N**_108_I_164_	2.3	3.6	0.772
Triple	C_**50**_**I**_51_**R**_59_**N**_108_I_164_	96.7	96.4	0.965
Triple	C_**50**_**I**_51_C_59_**N**_108_**L**_164_	0.5	0	1.000
Quadruple	C_**50**_**I**_51_**R**_59_**N**_108_**L**_164_	0.5	0	1.000
*Pfdhps*		n = 210	n = 107	
Wild	S_436_A_437_K_540_A_581_A_613_	0	0	N/A
Single	S_436_**G**_437_K_540_A_581_A_613_	0.5	2.8	0.221
Double	S_436_**G**_437_**E**_540_A_581_A_613_	88.1	62.6	**0.001**
Triple	**H**_436_**G**_437_**E**_540_A_581_A_613_	10.5	34.6	**0.001**
Triple	S_436_**G**_437_**E**_540_**G**_581_A_613_	1.0	0	0.793
*dhfr/dhps*		n = 209	n = 107	
Wild	C_50_N_51_C_59_S_108_I_164_/S_436_A_437_K_540_A_581_A_613_	0	0	N/A
Triple	C_50_**I**_51_C_59_**N**_108_I_164_/S_436_**G**_437_K_540_A_581_A_613_	0	1	1.000
Quadruple	C_50_**I**_51_C_59_**N**_108_I_164_/S_436_**G**_437_**E**_540_A_581_A_613_	2.4	2.8	1.000
Quadruple	C_50_**I**_51_**R**_59_**N**_108_I_164_/S_436_**G**_437_K_540_A_581_A_613_	0.5	1.9	0.553
Quintuple	C_50_**I**_51_**R**_59_**N**_108_I_164_/S_436_**G**_437_**E**_540_A_581_A_613_	85.2	59.8	**0.001**
Sextuple	C_50_**I**_51_**R**_59_**N**_108_I_164_/**H**_436_**G**_437_**E**_540_A_581_A_613_	10.1	34.6	**0.001**
Sextuple	C_50_**I**_51_C_59_**N**_108_**L**_164_/**H**_436_**G**_437_**E**_540_A_581_A_613_	0.5	0	1.000
Sextuple	C_50_**I**_51_**R**_59_**N**_108_**L**_164_/S_436_**G**_437_**E**_540_A_581_A_613_	0.5	0	1.000
Sextuple	C_50_**I**_51_**R**_59_**N**_108_I_164_/S_436_**G**_437_**E**_540_**G**_581_A_613_	1.0	0	0.791

P-values in bold indicate statistically significant differences in the prevalence of resistant markers between 2012 and 2017

*N/A* not applicable

### Temporal trend in the prevalence of *dhfr/dhps* haplotypes from 1996 to 2017

**Fig. 1:**
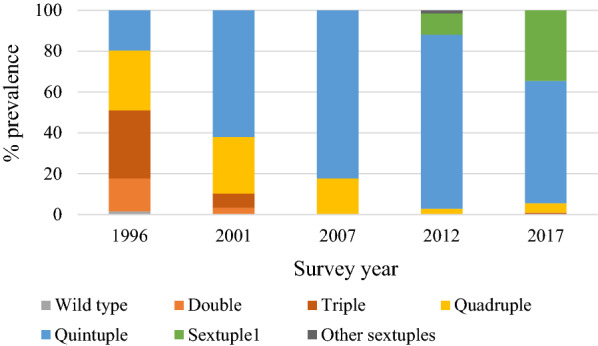
Temporal trend in the prevalence of combined *dhfr/dhps* haplotypes from 1996 to 2017. The data in 1996, 2001 and 2007 were extracted from previous publications [29, 30]

The prevalence of combined *dhfr/dhps* double, triple, and quadruple mutant haplotypes decreased significantly over time (all haplotypes p < 0.01), reflecting initially the increasing prevalence of the combined *dhfr/dhps* quintuple mutant haplotype (C_50_**I**_51_**R**_59_**N**_108_I_164_/S_436_**G**_437_**E**_540_ A_581_A_613_) from 19.7% in 1996 to 85.2% in 2012 (p < 0.01, Fig. 1). The prevalence of the quintuple mutant then declined slightly to 59.8% in 2017 as sextuple mutants increased. There was no *Pfdhps*-436H mutation found in 1996, 2001 and 2007 surveys in Asembo [29, 30]. The prevalence of sextuple mutant haplotypes in 2012 was 12.4% including 4 different sextuple haplotypes (Table 1). Of the four sextuple haplotypes, the haplotype C_50_**I**_51_**R**_59_**N**_108_I_164_/**H**_436_**G**_437_**E**_540_ A_581_A_613_ containing *Pfdhps*-436H appeared in 2012 and became the most common sextuple haplotype accounting for 10.5% in 2012 (Sextuple1, green bar in Fig. 1). This haplotype increased to 34.6% in 2017 (p < 0.01, green bar in Fig. 1 and Table 1).

### Prevalence of *Pfcrt* and *Pfmdr1* resistant SNPs and haplotypes in the 2012 and 2017 surveys

In 2012, the prevalence of *Pfcrt* SNPs was 17.3% for mutant *Pfcrt*-74I and 18.3% for both *Pfcrt*-75D/E and -76 T (Table 2). The prevalence of all three SNPs significantly decreased to 0.9% in 2017 (p = 0.001, Table 2). There were no mutations at codons 72 and 73 in either the 2012 or the 2017 samples. The prevalence of CQ-sensitive *Pfcrt*-K76 increased from 81.7% 2012 to 99.1% in 2017 (p = 0.001). Similarly, the wild haplotype C_72_V_73_M_74_N_75_K_76_ (CVMNK) also increased from 81.7% in 2012 to 99.1% in 2017 (p = 0.001), while the mutant haplotype C_72_V_73_**I**_74_**E**_75_**T**_76_ (CV**IET**) decreased from 10.6% to 0.9% (1/110) (p = 0.003), and the mutant haplotype C_72_V_73_**I**_74_**D**_75_**T**_76_ (CV**IDT**) from 6.7% to 0.0% (p = 0.013).

**Table 2:** Prevalence of *Pfcrt and Pfmdr1* mutant *S*NPs and haplotypes between 2012 and 2017

Gene	SNP	Percent in 2012	Percent in 2017	P value
***Pfcrt***		n = 208	n = 110	
	C72S	0	0	N/A
	V73V	100	100	N/A
	M74I	17.3	0.9	**0.001**
	N75D/E	18.3	0.9	**0.001**
	K76T	18.3	0.9	**0.001**
*Pfmdr1*		n = 207	n = 111	
	N86Y	10.2	0	**0.001**
	Y184F	52.2	55.9	0.61
	S1034C	0	0	N/A
	N1042D	0	0	N/A
	D1246Y	13.5	9.9	0.448
Mutation number	**Haplotype**	**Percent in 2012**	**Percent in 2017**	**P value**
*Pfcrt*		n = 208	n = 110	
Wild	C_72_V_73_M_74_N_75_K_76_	81.7	99.1	**0.001**
Double	C_72_V_73_M_74_**D**_75_**T**_76_	1.0	0	0.774
Triple	C_72_V_73_**I**_74_**E**_75_**T**_76_	10.6	0.9	**0.003**
Triple	C_72_V_73_**I**_74_**D**_75_**T**_76_	6.7	0	**0.013**
*Pfmdr1*		n = 207	n = 111	
Wild	N_86_Y_184_S_1034_N_1042_D_1246_	36.7	40.5	0.562
Single	N_86_**F**_184_S_1034_/N_1042_/D_1246_	45.4	49.6	0.557
Single	N_86_Y_184_S_1034_N_1042_**Y**_1246_	3.4	3.6	1.000
Single	**Y**_86_Y_184_S_1034_N_1042_D_1246_	2.9	0	0.168
Double	N_86_**F**_184_S_1034_N_1042_**Y**_1246_	4.4	6.3	0.622
Double	**Y**_**86**_Y_184_S_1034_N_1042_**Y**_1246_	4.8	0	**0.043**
Double	**Y**_86_**F**_184_S_1034_N_1042_D_1246_	1.5	0	**0.055**
Triple	**Y**_86_**F**_184_S_1034_N_1042_**Y**_1246_	1.0	0	0.768

*N/A* is not applicable

P value in bold indicates significant or marginal differences of the prevalence of resistant markers between 2012 and 2017

The prevalence of mutant SNPs *Pfmdr1-*86Y,-184F, and -1246Y was 10.2%, 52.2% and 13.6%, respectively in 2012, and zero, 55.9% and 9.9%, respectively in 2017 (Table 2) with a significant decline of *Pfmdr1*-86Y in 2017 (p = 0.001). No samples carried mutations for *Pfmdr1-*1034 or *Pfmdr1*-1042 in either the 2012 or the 2017 samples. There were no statistically significant differences in the prevalence of wild haplotype N_86_Y_184_S_1034_N_1042_D_1246_ (NYSND) or the major mutant haplotype N_86_**F**_184_S_1034_N_1042_D_1246_ (N**F**SND) between 2012 and 2017 (Table 2). However, the prevalence of mutant haplotypes **Y**_86_Y_184_S_1034_N_1042_**Y**_1246_ (**Y**YSN**Y**) decreased significantly (p = 0.043) and **Y**_86_**F**_184_S_1034_N_1042_D_1246_ (**YF**SND) marginally (p = 0.055).

### Temporal trend in prevalence of *Pfcrt* and *Pfmdr1* SNPs and haplotypes from 1996 to 2017

**Fig. 2:**
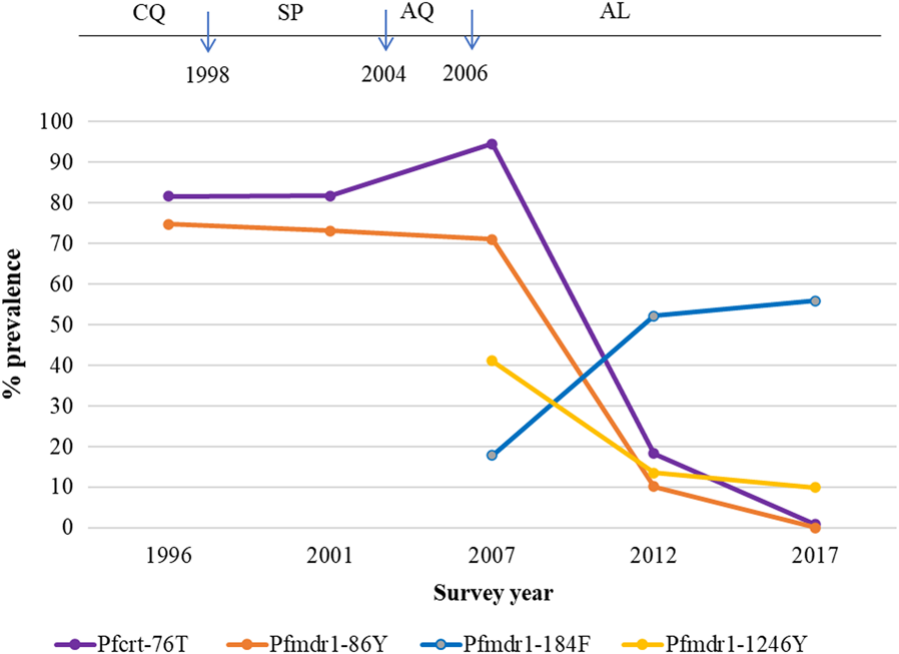
Temporal trends in the prevalence of *Pfcrt* and *Pfmdr1* resistant SNP markers over years. The data in 1996, 2001 and 2007 were extracted from publications [29, 30]

Since previous studies using samples from the 1996 and 2001 surveys by this research group in the same area only tested the key markers of *Pfcrt* K76T and *Pfmdr1* N86Y by real-time PCR [30], these were the only SNPs evaluated in the temporal trends from 1996 to 2017; for the temporal trends from 2007 to 2017, other SNPs and haplotypes of *Pfcrt* and *Pfmdr 1* were included*.* The prevalence of mutant *Pfcrt-*76 T SNP was 81.6% and 81.8% in 1996 and 2001 and increased to 94.6% in 2007. Thereafter, the prevalence declined sharply to 18.3% in 2012 and 0.9% in 2017 (p < 0.01, Fig. 2). The wild type *Pfcrt-*K76 significantly increased from 18.4% to 99.1% from 1996 to 2017 a period of over 20 years (p < 0.01). Changing trends were also observed for *Pfmdr1* codons 86, 184 and 1246 over years. The prevalence of mutant *Pfmdr1*-86Y slowly decreased to 71.0% in 2007, then declined to zero in 2017 (p < 0.01, Fig. 2). The prevalence of mutant *Pfmdr1*-1246Y decreased from 41.1% in 2007 to 9.9% in 2017 (p < 0.01). In contrast, mutant *Pfmdr1*-184F increased from 17.9% in 2007 to 55.9% in 2017 (p < 0.01, Fig. 2).

**Fig. 3:**
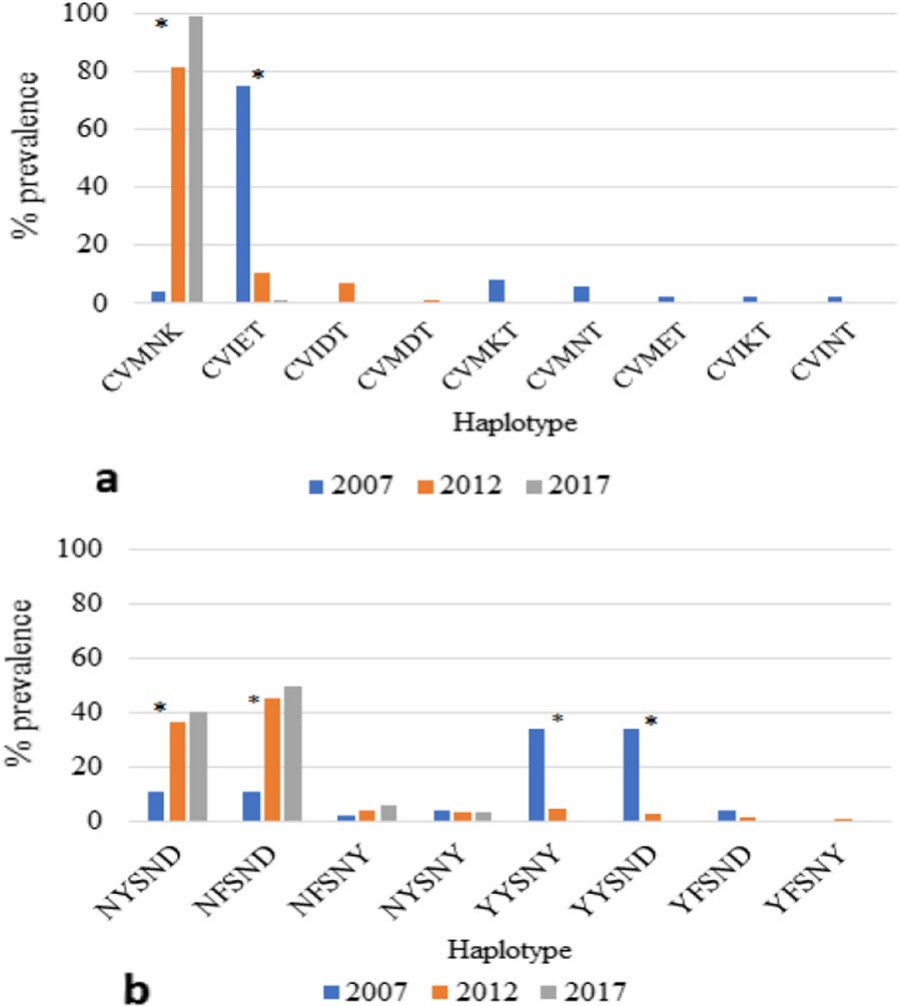
Temporal trend in the prevalence of *Pfcrt* and *Pfmdr1* haplotypes from 2007 to 2017. **3a** The construction of *Pfcrt* haplotype includes codons 72, 73, 74, 75, 76. Codons at 72 and 73 were wild type in all samples tested in this paper. CVMNK, the first label, is the wild haplotype of *Pfcrt* and remaining labels are all mutant haplotypes. **3b** The construction of *Pfmdr1* haplotype includes codons 86, 184, 1034, 1042, 1246. Codons at 1034 and 1042 were wild types in all samples tested in this paper. NYSND, the first label, is the wild haplotype of *Pfmdr1* and remaining labels are all mutant haplotypes. Symbols “*” indicate statistical significance in prevalence of haplotypes over time (p < 0.01). The data in 2007 were extracted from publications [30]

Figure 3a shows the decline of major mutant haplotype C_72_V_73_**I**_74_**E**_75_**T**_76_ (CV**IET**) of *Pfcrt* from 75.0% to 0.9% from 2007 to 2017 (p < 0.01) while the wild haplotype C_72_V_73_M_74_N_75_K_76_ (CVMNK) of *Pfcrt* increased from 4.0% of 2007 to 99.1% in 2017 (p < 0.01). The parasites carrying *Pfmdr1* N_86_F_184_S_1034_N_1042_D_1246_ (N**F**SND) haplotype (containing a mutant SNP at codon 184F), possibly associated with reduced lumefantrine susceptibility, increased from 11.0% in 2007 to 49.6% in 2017 (p < 0.01, Fig. 3b). There was a significant increase in the prevalence of wild haplotype N_86_Y_184_S_1034_N_1042_D_1246_ (NYSND) from 11.1% to 40.5% (p < 0.01) as well as a decrease in the prevalence of two other mutant haplotypes, **Y**_86_Y_184_S_1034_N_1042_**Y**_1246_ (**Y**YSN**Y**) from 34.0% to 4.8% (p < 0.01) and **Y**_86_Y_184_S_1034_N_1042_D_1246_ (**Y**YSND) from 34.0% to 2.9% (p < 0.01), from 2007 to 2017.

### Artemisinin resistance marker *Pfk13* gene at the 2012 and 2017 surveys

There were no artemisinin-associated resistant *Pfk13* mutations at the nine validated codons or other candidate codons listed by the WHO (2018) [38] in any of the samples tested in the 2012 and 2017 surveys in the Asembo area. Two samples from the 2017 survey had the *Pfk13* synonymous mutation at codons 504 and 510, respectively.

### Comparison of drug resistance molecular markers in parasites from mosquitoes and humans in 2012 survey

**Fig. 4:**
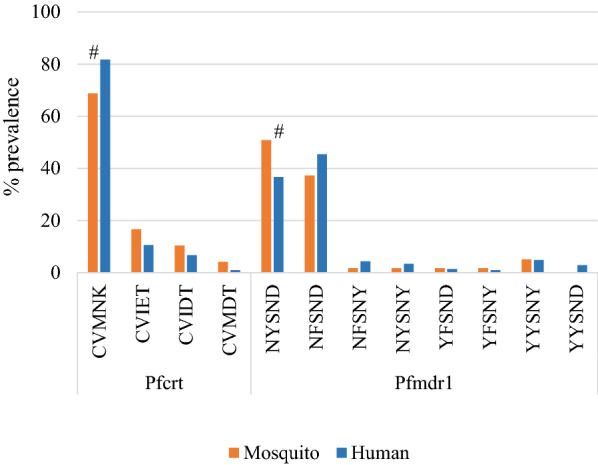
Comparison of prevalence of *Pfcrt* and *Pfmdr1* haplotypes between mosquito oocyst and human blood samples. CVMNK and NYSND are wild haplotypes for *Pfcrt* and *Pfmdr1*, respectively. The remaining haplotypes have at least one mutant allele. Symbols “#” indicate 0.05 ≤ p < 0.10

A total of 66 mosquitoes collected in 2012 were found to be oocyst positive and analysed for the frequency of molecular markers of drug resistance. Among oocyst-positive mosquitoes, *An.s funestus* was the predominant species (87.9%), compared to *An. gambiae *s.s*.* (6.1%) and *An. arabiensis* (4.5%). Species identification by PCR failed for one mosquito sample. There were marginal differences in the prevalence of three drug resistant SNPs in the parasites from mosquitos compared to human, with a lower frequency of *Pfdhps-*436H (p = 0.059) and a higher frequency of *Pfcrt-*75D/E, − 76 T in mosquito oocysts (p = 0.051) (Table 3). The prevalence of parasites carrying wild haplotype *Pfcrt* C_72_V_73_M_74_N_75_K_76_ (CVMNK) was lower in mosquitoes than in human blood with marginal statistical significance (p = 0.051, Fig. 4). There were eight *Pfmdr1* haplotypes of parasites in humans but seven haplotypes in mosquitoes with **Y**_86_Y_184_S_1034_N_1042_D_1246_ (**Y**YSND) not observed in mosquitoes. The prevalence of *Pfmdr1* wild haplotype N_86_Y_184_S_1034_N_1042_D_1246_ (NYSND) was marginally higher in mosquito oocysts than in human blood samples (p = 0.070, Fig. 4). Since mosquitoes were collected from a single village while sampling of parasites from humans was 54 villages across the entire HDSS area, additional analyses were conducted to assess whether the observed marginal differences in drug resistance markers were the result of biases due to the limited geographic sampling area for mosquitoes. The distance from the household of each human sampled for parasite infection to the centroid of the village where mosquitoes were collected was calculated. Households were then categorized as < 7 km, < 14 km and < 27 km from the centroid of the village where mosquitoes were collected. The initial selection of 7 km distance was based on the upper limit of a basic mosquito reproductive unit as assessed by kinship analysis of *An. gambiae s.s.* using microsatellite loci in this area [41]. Comparisons of the frequency of drug resistance SNP markers between human blood samples and oocysts in mosquitoes showed similar trends with similar marginal p-values within the different distance categories. These analyses suggest that there was no substantial variation in the frequency of molecular markers of drug resistance across the investigation and that the marginal differences observed between parasites from humans versus mosquitoes were not substantially biased by the limited geographic sampling area for mosquitoes (see Additional file 3).

**Table 3.** Comparison of prevalence of drug resistance molecular markers in parasites between mosquitoes and human hosts

Gene	SNP	Mosquitoes	Human blood	P value
		Prevalence (95% CI)	Prevalence (95% CI)	
***Pfdhfr***		n = 62	n = 212	
	N51I	98.4 (91.3, 100)	100 (98.3, 100)	0.226
	C59R	95.2 (86.5, 99.0)	97.2 (93.9, 99.0)	0.689
	S108N	98.4 (91.3, 100)	100 (98.3, 100)	0.226
	I164L	0 (0, 5.8)	0.9 (0.1, 3.4)	1.000
***Pfdhps***		n = 58	n = 210	
	S436H	1.7 (0, 9.2)	10.5 (6.7, 15.4)	**0.059**
	A437G	98.3 (90.8, 100)	100 (98.3, 100)	0.216
	K540E	98.3 (90.8, 100)	99.5 (97.4, 100)	0.387
	A581G	1.7 (0, 9.2)	1.0 (0.1, 3.4)	1.000
***Pfcrt***		n = 48	n = 208	
	M74I	27.1 (15.3, 41.9)	17.3 (12.4, 23.2)	0.153
	N75D/E	31.3 (18.7, 46.3)	18.3 (13.3, 24.2)	**0.051**
	K76T	31.3 (18.7, 46.3)	18.3 (13.3, 24.2)	**0.051**
***Pfmdr1***		n = 65	n = 207	
	N86Y	9.2 (3.5, 19.0)	10.1(6.4, 15.1)	1.000
	Y184F	43.1 (30.9, 56.0)	52.2 (45.1, 59.2)	0.255
	D1246Y	10.1 (4.8, 22.6)	13.5 (9.2, 19.0)	0.830

P values in bold indicate marginal differences (0.05 ≤ p < 0.10).

## Discussion

This time series analysis of changes in the prevalence of molecular markers of anti-malarial drug resistance over 20 years in western Kenya showed a near fixation of the *Pfdhfr*-51I, -59R, -108 N and *Pfdhps*-437G, -540E resistance markers and a significant increase in the sextuple mutant haplotype containing the *Pfdhps*-436H allele between 2012 and 2017. In contrast, a near complete return of the wild type haplotype of *Pfcrt* and *Pfmdr1* was observed in 2017, 19 years after the withdrawal of CQ and 12 years after the wide-scale uptake of AL in the study area. No validated or candidate mutations in the *Pfk13* propeller domain associated with artemisinin resistance were found in 2012 or 2017 based on WHO 2018 standards [38]. Marginal differences in the prevalence of *Pfdhps-*436H and *Pfcrt*-76 T were observed between parasites from human blood samples and mosquitoes.

Despite the discontinuation of SP as first line treatment for uncomplicated malaria in the general population in 2004, the prevalence of combined SP *dhfr/dhps* quintuple mutant haplotype (C_50_**I**_51_**R**_59_**N**_108_I_164_/S_436/_**G**_437_**E**_540_A_581_A_613_) steadily increased to 85.2% in 2012, then decreased in 2017 to 59.8%. However, this decrease was largely due to an increased prevalence of sextuple mutant haplotype (C_50_**I**_51_**R**_59_**N**_108_I_164_/**H**_436_**G**_437_**E**_540_A_581_A_613_), resulting from the rise in *Pfdhps*-436H allele in the Asembo area from 0.0% in 2007 [30] to 10.5% in 2012 and 34.6% in 2017. This trend was consistent with other studies in western Kenya reporting that *Pfdhps*-436H allele was undetectable in 2005, 14.1% between 2010 and 2013 in samples collected from children in the Bondo District Hospital and the Siaya District Hospital of western Kenya [42], and 28.0% in 2018 in children enrolled at the Siaya County Referral Hospital [43]. In addition, the *Pfdhps*-436H mutation was observed at 3.8% in pregnant women in 2008 to 2009 in western Kenya [44]. Taken together, it is plausible that the increase in prevalence of *Pfdhps*-436H mutant allele and sextuple mutant haplotype in the parasites in general population might originate from a slow expansion of such mutant parasites from the pregnant women on IPTp-SP. It is also possible that cross-resistance to other antifolate drugs, such as cotrimoxazole (CTX) commonly used as prophylaxis against opportunistic infection in HIV infected individuals or as treatment for other bacterial infections in general populations [45–47], could select for the *Pfdhps-*436H in this area. However, a recent trial has shown that CTX does not select for SP resistance [48]. Although the effect of the *Pfdhps*-436H mutation on SP resistance is not yet known, the progressive increase of this mutant allele indicates a need to closely monitor its impact on the effectiveness of IPTp with SP in western Kenya.

The temporal trend analysis showed that the prevalence of *Pfcrt*-76 T mutants increased from 81.0% in 2001 to a peak prevalence (94.6%) in 2007, during which AQ, which may exert similar selective pressure as CQ, was still used in this area [29, 30, 42]. This was then followed by the sharp decline in resistance marker *Pfcrt-76 T* from 94.6% in 2007 to 18.3% in 2012 and 0.9% in 2017 and a marked increase in the CQ-sensitive haplotype (C_72_V_73_M_74_N_75_K_76_) from 4.0% in 2007 to 81.7% in 2012 and to 99.1% in 2017. This reversal of *Pfcrt* from CQ-resistant to CQ-sensitive genotype, after long-term withdrawal of CQ (19 years), has been reported previously in other countries [49], as well as in coastal Kenya and other areas of western Kenya [50]. The reduced prevalence of CQ-resistant genotypes reflects a fitness cost to the parasites after long-term withdrawal of CQ and less use of AQ [51].

However, the strong temporal trend, following the widespread introduction of artemether-lumefantrine as first-line treatment in the study area in 2006, suggests that counter-selection pressure from lumefantrine on this gene may have also played an important role. AL and CQ or AQ exert opposite selection pressure on the *Pfcrt* genotypes as AL selects *Pfcrt*-K76 while AQ and CQ select *Pfcrt*-76 T [52, 53]. Overall, the reversal of *Pfcrt* to CQ sensitive genotype may indicate the option of re-introducing CQ as a component of ACT using a strategy of negative cross-resistance or as part of a triple-drug ACT combination [54–56] though there are debates on this re-introduction of CQ.

CQ and AQ also select for *Pfmdr1-*86Y and -1246Y SNPs, while AL selects for the *Pfmdr1-*N86, -184F, and -D1246 alleles [52]. Previous studies have shown a strong selection of wild *Pfmdr1-*N86 in re-infection after treatment with AL [57, 58] and the increase of *Pfmdr1-*N86 is associated with reduced susceptibility to AL treatment [52]. It has been reported that the selection of wild *Pfmdr1-*N86 is mediated by lumefantrine drug pressure in vitro [59, 60]. The counter-selection of wild type *Pfmdr1-*N86 has also been observed in use of other antimalarials, such as mefloquine vs CQ/AQ [55, 61]. In addition, mutant SNP *Pfmdr1-*184F was considered to be under selection by AL [36] and was associated with reduced lumefantrine susceptibility in vivo and ex vivo [18, 62]. The *Pfmdr1* haplotype N_86_F_184_D_1246_ was significantly associated with AL treatment failures in Nigerian children [15]. The temporal analysis in this study showed that wild type *Pfmdr1*-N86 increased across years from 25.2% in 1996 at baseline to 100% in 2017 and mutant SNP *Pfmdr1*-184F increased in 2012 and 2017 in the Asembo area. Accordingly, both wild haplotype N_86_Y_184_S_1034_N_1042_D_1246_ and the haplotype carrying 184F mutant codon, N_86_**F**_184_S_1034_N_1042_D_1246_ steadily increased over years. These results are consistent with previous findings in other areas of western Kenya [63, 64] and other African countries [65, 66]. There are two combined effects for this phenomenon. First, the increase in wild haplotype N_86_Y_184_S_1034_N_1042_D_1246_ indicates returning to CQ sensitivity by changes from *Pfmdr1*-86Y to *Pfmdr1*-N86 and from *Pfmdr1*-1246Y to *Pfmdr1*-D1246 (Fig. 2) after withdrawal of CQ or AQ in the area [30, 53]. Second, counter-selection of wild *Pfmdr1-*N86, -D1246 and selection of mutant *Pfmdr1-*184F by use of AL [15] over a decade could result in the increase of mutant N_86_**F**_184_S_1034_N_1042_D_1246_ haplotype.

AL was officially recommended as first-line ACT for treatment of uncomplicated malaria in 2004 with widespread implementation since the end of 2006 [5] in Kenya. In the current 2012 and 2017 community surveys, no artemisinin-associated drug resistant mutations were found after a decade of AL use in the Asembo area based on WHO 2018 standard [38]. These molecular *Pfk13* findings were consistent with the previous report in a 2016–2017 TES conducted in Siaya county of western Kenya [67]. There were no overt signs of delayed parasite clearance on day 3 [Nelli Westercamp et al. unpublished data] and no mutations associated with artemisinin resistance [67]. However, the PCR-corrected adequate sustained clinical and parasitological response (ACPR) for AL was 88.5% on day 28 while that for dihydroartemisinin–piperaquine remained high at day 42 in the same TES study [Nelli Westercamp et al. unpublished data]. This may suggest parasite sensitivity to the Artemisinin partner drug, lumefantrine is waning. Although the prevalence of *Pfmdr1*-184F mutations was high, their association with treatment failure was not statistically significant [67]. These results are poorly understood and further investigation of association between *Pfmdr1*-184F and lumefantrine is warranted in Kenya. Moreover, *Pfk13* mutations associated with delayed parasite clearance have been locally emerged in Rwanda (*Pfk13* R561H SNP) and Uganda (*Pfk13* A675V and C469Y SNPs), respectively [68, 69]. Therefore, continued monitoring of *Pfk13* molecular markers is necessary in African countries including Kenya for early detection of AL drug resistance.

The present study showed a marginally higher prevalence of mutant *Pfcrt*-76 T SNP in mosquito oocysts compared to human blood samples, suggesting that *Pfcrt* mutation-bearing parasites might be more transmissible in this setting of western Kenya. In contrast, the lower prevalence of *Pfdhps-*436H mutant in mosquito oocysts compared to humans was observed in this study, indicating that there could be a fitness cost in *An. funestus* mosquitoes for transmission of the *Pfdhps*-436H mutation bearing parasites in the Asembo area. Differences in mutation profiles between human and mosquito hosts and among species of mosquitoes have been documented previously. For instance, a previous study in a high transmission area of Zambia has shown that the prevalence of resistant SNP of cycloguanil (Proguanil metabolite), *Pfdhfr*-108 T, is high in human blood (> 90.0%), but very rare in the midgut of *An. arabiensis* [26]. In Southern Zambia, there was a lower prevalence of mutant *Pfcrt*-76 T SNP bearing parasites in *An. arabiensis* mosquitoes compared to human blood samples [24]. However, in Uganda significantly higher prevalence of mutant *Pfcrt*-76 T and *Pfmdr1*-86Y was observed in *An. gambiae* complex mosquitoes than in human blood [25]. Another study conducted in Tanzania showed that *An. arabiensis* consistently carried a lower proportion of *Pfcrt-*76 T mutant than both *An. funestus* and *An. gambiae *s.s. from the salivary glands [27]. These studies highlight the heterogeneity in transmission of different markers of anti-malarial drug resistance, which is likely affected by the species of mosquito, the individual markers of drug resistance and the transmission settings. These studies also suggest that mosquito-parasite interactions have the potential for either accelerating or slowing the transmission of drug resistance, although the mechanisms involved are still unknown. Further studies are needed to understand how different mosquito vectors transmit parasites with different drug resistance markers in different transmission settings.

## Limitations

There were a few limitations in the present study. First, the sample size for genotyping drug resistance markers of parasites in mosquito oocysts was small, which could affect statistical power for detecting statistically significant differences between the two hosts. Second, the mosquitoes collected were not from the same houses where the human blood samples were collected, and there was a slight difference in the months between the collection of mosquitoes (May to September) and human blood (June to August). This limited us from conducting true pair-wise comparisons. However, the marginal differences in prevalence of *Pfdhps*-436H and *Pfcrt*-76 T mutant SNPs at population level between two hosts observed in this study deserve further investigation.

## Conclusions

This study showed an increased prevalence of *dhfr/dhps* resistant markers over 20 years with the emergence of *Pfdhps-*436H mutant a decade ago in Asembo. CQ-sensitive haplotype reached nearly 100% in 2017, a reversal from CQ-resistant to CQ-sensitive genotype at the *Pfcrt* gene after 19 years of the withdrawal of CQ. No *Pfk13* markers associated with artemisinin resistance were detected, but the increasing prevalence of *Pfmdr1* N_86_**F**_184_S_1034_N_1042_D_1246_ haplotype was observed. The differences in prevalence of *Pfdhps*-436H and *Pfcrt*-76 T SNPs between two hosts and the role of mosquitoes in the transmission of drug resistant parasites require further investigation. Continued monitoring of the molecular resistance markers in this area is imperative to guide anti-malarial policy and delay the emergence and spread of drug resistance.

## Disclaimer

The findings and conclusions in this report are those of the authors and do not necessarily represent the official position of the Centers for Disease Control and Prevention.

## Supplementary Information

**Additional file 1: Table S1.** Primer sequences and reaction conditions for nested PCRs of *Pfdhfr* and *Pfdhps.*

**Additional file 2: Table S2.** Primers used for PCR and sequencing for *Pfcrt*, *Pfmdr1* and *Pfk13* genes.

**Additional file 3: Table S3.** Comparison in prevalence of drug resistance SNPs of parasites between mosquitoes and human blood samples collected by three categories of distances.

## Data Availability

The molecular dataset used in this study is available and can be shared upon reasonable request to the corresponding authors.

## Abbreviations

ACPR: Adequate sustained clinical and parasitological response
ACT: Artemisinin-based combination therapy
AL: Artemether-lumefantrine
AQ: Amodiaquine
DBS: Dried blood spot
CQ: Chloroquine
CTX: Cotrimoxazole
DHA: Dihydroartemisinin
HDSS: Health and demographic surveillance system
*Pfdhfr*: *Plasmodium falciparum* Dihydrofolate reductase
*Pfdhps*: *Plasmodium falciparum* Dihydropteroate synthase
ITN: Insecticide-treated net
IPTp: Intermittent preventive treatment during pregnancy
LLIN: Long lasting insecticide-treated bed nets
PCR: Polymerase chain reaction
*Pfcrt*: *Plasmodium falciparum* Chloroquine resistance transporter gene
*Pfk13*: *Plasmodium falciparum* Kelch 13
*Pfmdr1*: Multi-drug resistance protein 1
SNP: Single nucleotide polymorphism
SP: Sulfadoxine-pyrimethamine
TES: Therapeutic efficacy study
WHO: World Health Organization
