# Supplementary material for: Temporal trends in molecular markers of drug resistance in Plasmodium falciparum in human blood and profiles of corresponding resistant markers in mosquito oocysts in Asembo, western Kenya
Source: Malar J. 2022 Sep 13;21:265. doi: 10.1186/s12936-022-04284-6 (PMC9472345; doi:10.1186/s12936-022-04284-6)
Supplement: Supplementary file 1 — Additional file 1: Table S1. Primer sequences and reaction conditions for nested PCRs of Pfdhfr and Pfdhps. [file 12936_2022_4284_MOESM1_ESM.docx]

**Additional file 1: Table 1. Primer sequences and reaction conditions for nested PCRs of *Pfdhfr* and *Pfdhps***

| **Gene** | **Primer** | **Primer sequence (5’---> 3’)** | **Product size (bp)** | **PCR cycling condition** |
| --- | --- | --- | --- | --- |
| ***Pfdhfr*** | **Primary PCR** |  |  |  |
|  | dhfr-PF1 | ATGATGGAACAAGTCTGCGAC | 700 | 95°C x 5 min; 40 cycles of 94°C x 30 sec, 45°C x 45 sec, 65°C x 45 sec; 72°C x 5 min |
|  | dhfr-PR1 | ACATTTTATTATTCGTTTTCT |  |  |
| ***Pfdhfr*** | **Secondary PCR** |  | |  |
|  | dhfr-NF1 | GCGACGTTTTCGATATTTATGC | 543 | 95°C x 5 min; 35 cycles of 94°C x 30 sec, 45°C x 30 sec, 72°C x 45 sec; 72°C x 5 min |
|  | dhfr-NR1 | CACATTCATATGTACTATTT |  |  |
| ***Pfdhps*** | **Primary PCR** |  | |  |
|  | dhps-PF2 | GGAATATTAAATGTTAATTATGATTCT | 947 | 95°C x 5 min; 40 cycles of 94°C x 30 sec, 45°C x 45 sec, 72°C x 45 sec; 72°C x 5 min |
|  | dhps-PR2 | ACCTGAAAAGAAATACATAA |  |  |
| ***Pfdhps*** | **Secondary PCR** |  | |  |
|  | dhps-NF2 | TGTTCAAAGAATGTTTGAAATGA | 694 | 95°C x 5 min; 35 cycles of 94°C x 30 sec, 42°C x 30 sec, 72°C x 45 sec; 72°C x 5 min |
|  | dhps-NR2 | GTGTGATTTGTCCACAATAT |  |  |
|  | dhps-NR3 | CAACTAAATCATTATCAACACATT |  | Internal sequencing primer only |

Note: for both of *Pfdhfr* and *Pfdhps* primary PCR, a 25-ul reaction contained 12.5 µl 2× master mix, 1 µl (400 nM) each primer, 2 µl DNA template and 8.5 µl PCR-grade water. For secondary PCR, reaction system is the same except for template with 2 µl primary PCR products for both genes.
