# Supplementary material for: Temporal trends in molecular markers of drug resistance in Plasmodium falciparum in human blood and profiles of corresponding resistant markers in mosquito oocysts in Asembo, western Kenya
Source: Malar J. 2022 Sep 13;21:265. doi: 10.1186/s12936-022-04284-6 (PMC9472345; doi:10.1186/s12936-022-04284-6)
Supplement: Supplementary file 2 — Additional file 2: Table S2. Primers used for PCR and sequencing for Pfcrt, Pfmdr1 and Pfk13 genes. [file 12936_2022_4284_MOESM2_ESM.docx]

**Additional file 2: Table 2. Primers used for PCR and sequencing for *Pfcrt*, *Pfmdr1* and *Pfk13* genes**

| **Primer** | **Sequence (5’---> 3’)** | **Purpose** | **Reference** |
| --- | --- | --- | --- |
| Pfcrt-PF1 | AGCAAAAATGACGAGCGTTATAG | Primary PCR for codon 72-76 | 35 |
| Pfcrt-PR1 | ATTGGTAGGTGGAATAGATTCTC | Primary PCR for codon 72-76 |  |
| Pfcrt-NSF2 | TTTTTCCCTTGTCGACCTTAAC | Nested & sequencing |  |
| Pfcrt-NSR2 | AGGAATAAACAATAAAGAACATAATCATAC | Nested & sequencing |  |
| Mdr1-PF1 | CCGTTTAAATGTTTACCTGCAC | Primary PCR for codon 86-184 | 36 |
| Mdr1-PR1 | TGGGGTATTGATTCGTTGCAC | Primary PCR for codon 86-184 |  |
| Mdr1-NSF1 | GTATGTGCTGTATTATCAGGAG | Nested & Sequencing for 86-184 |  |
| Mdr1-NSR1 | AGCCTCTTCTATAATGGACATG | Nested & Sequencing for 86-184 |  |
| Mdr1-PF2 | GCATTTAGTTCAGATGATGAAATG | Primary PCR for codon 1034-1246 | 36 |
| Mdr1-PR2 | CCATATGGTCCAACATTTGTATC | Primary PCR for codon 1034-1246 |  |
| Mdr1-SF2 | TATGCATACTGTTATTAATTATGG | Sequencing for 1034-1042 |  |
| Mdr1-SR2 | TTTTGCATTTTCTGAATCTCCTT | Sequencing for 1034, 1042 | This paper |
| Mdr1-SF3 | GCAATCGTTGGAGAAACAGG | Sequencing for codon 1246 |  |
| Mdr1-SR3 | TTCGATAAATTCATCTATAGCAG | Sequencing for codon 1246 | 36 |
| K13-PF1 | GCAAATAGTATCTCGAAT | Primary PCR | 37 |
| K13-PR1 | CTGGGAACTAATAAAGAT | Primary PCR |  |
| K13-NSF2 | GATAAACAAGGAAGAATATTCT | Nested PCR & sequencing |  |
| K13-NSR2 | CGGAATCTAATATGTTATGTTCA | Nested PCR & sequencing |  |

**Additional file 2: PCR reaction systems for *Pfcrt*, *Pfmdr1* and *Pfk13* genes:**

Prepare the primary PCR master mix as below for *Pfcrt*:

| **Reagents** | **1 PCR (25-µl reaction)** | **110 PCR** |
| --- | --- | --- |
| 2 X Promega Master Mix | 12.5 µl | 1375 µl |
| Pfcrt-PF1 (10 µM) | 0.5 µl | 55 µl |
| Pfcrt-PR1 (10 µM) | 0.5 µl | 55 µl |
| Nuclease-free water | 9.5 µl | 1045 µl |
| **Total** | 23 µl | 1. µl |

PCR template: 2 µl extracted DNA for each 25-ul reaction.

PCR cycling condition:

| **One Cycle (hold)** |  |
| --- | --- |
| 95°C | 5 minutes |
| **30 PCR cycles** |  |
| 95°C | 30 seconds |
| 59°C | 30 seconds |
| 72°C | 30 seconds |
| **One cycle (hold)** |  |
| 72°C | 10 minutes |
| 4°C | ∞ |

Preparation of *Pfcrt* secondary PCR master mix:

| **Reagents** | **1 PCR reaction** | **110 PCR reaction** |
| --- | --- | --- |
| 2 X Master Mix | 12.5 µl | 1375 µl |
| Pfcrt-NSF2 (10 µM) | 0.5 µl | 110 µl |
| Pfcrt-NSR2 (10 µM) | 0.5µl | 110 µl |
| Nuclease-free water | 9.5 µl | 1045 µl |
| **Total** | 23 µl | 1. µl |

PCR template: 2 µl primary PCR product for each 25-ul reaction.

PCR cycling condition:

| **One cycle** |  |
| --- | --- |
| 95°C | 5 minutes |
| **25 PCR cycles** |  |
| 95°C | 30 seconds |
| 56°C | 30 seconds |
| 72°C | 30 seconds |
| **One cycle** |  |
| 72°C | 10 minutes |
| 4°C | ∞ |

**Amplification of *Pfmdr1* gene fragment for codon 86 & 184 (Region 1):**

| **Reagents** | **1 PCR** | **110 PCR** |
| --- | --- | --- |
| 2X promega Master Mix | 12.5 µl | 1375 µl |
| MDR1-PF1 (10 µM) | 0.5 µl | 55 µl |
| MDR1-PR1 (10 µM) | 0.5 µl | 55 µl |
| Nuclease-free water | 9.5 µl | 1045 µl |
| **Total** | 23 µl | 1. µl |

Add 2 µl extracted DNAs in each reaction.

PCR cycling condition:

| **One cycle** |  |
| --- | --- |
| 95°C | 5 minutes |
| **30 PCR cycles** |  |
| 95°C | 1 minute |
| **57**°C | 1 minute |
| 72°C | 1 minute |
| **One cycle** |  |
| 72°C | 10 minutes |
| 4°C | ∞ |

***Pfmdr1* 86-184 secondary PCR:**

| **Reagents** | **1 PCR reaction** | **110 PCR reaction** |
| --- | --- | --- |
| 2 X Master Mix | 12.5 µl | 1375 µl |
| MDR1-SF1 (10 µM) | 0.5 µl | 55 µl |
| MDR1-SR1 (10 µM) | 0.5 µl | 55 µl |
| Nuclease-free water | 9.5 µl | 1045 µl |
| **Total** | 23 µl | 1. µl |

Add primary PCR product 2 µl into each reaction.

PCR cycling condition:

| **One cycle** |  |
| --- | --- |
| 95°C | 5 minutes |
| **30 PCR cycles** |  |
| 95°C | 30 seconds |
| **55**°C | 30 seconds |
| 72°C | 45 seconds |
| **One cycle** |  |
| 72°C | 10 minutes |
| 4°C | ∞ |

**Amplification of *Pfmdr1* gene fragments for codon 1034-1246 (Region 2 & 3):**

| 2X Promega Master Mix | 12.5 µl | 1375 µl |
| --- | --- | --- |
| MDR1-PF2 (10 µM) | 0.5 µl | 55 µl |
| MDR1-PR2 (10 µM) | 0.5 µl | 55 µl |
| Nuclease-free water | 9.5 µl | 1045 µl |
| **Total** | 23 µl | 1. µl |

Add 2 µl extracted DNAs in each reaction.

PCR cycling condition:

| **One cycle** |  |
| --- | --- |
| 95°C | 5 minutes |
| **30 PCR cycles:** |  |
| 95°C | 1 minute |
| **56**°C | 1 minute |
| 65°C | 1 minute |
| **One cycle** |  |
| 72°C | 10 minutes |
| 4°C | ∞ |

**Prepare the secondary PCR master mix for codon 1034 & 1042 (Region 2) as below:**

| **Reagents** | **1 PCR reaction** | **110 PCR reaction** |
| --- | --- | --- |
| 2 X Master Mix | 12.5 µl | 1375 µl |
| MDR1-SF2 (10 µM) | 0.5 µl | 55 µl |
| MDR1-SR2 (10 µM) | 0.5 µl | 55 µl |
| Nuclease-free water | 9.5 µl | 1045 µl |
| Total | 23 µl | 1. µl |

Add primary PCR product 2 µl into the reaction well.

PCR cycling condition:

| **One cycle** |  |
| --- | --- |
| 95°C | 5 minutes |
| **25 PCR cycles:** |  |
| 95°C | 30 seconds |
| **54**°C | 45 seconds |
| 72°C | 45 seconds |
| **One cycle** |  |
| 72°C | 10 minutes |
| 4°C | ∞ |

**Prepare the secondary PCR master mix for codon 1246 (Region 3) as below:**

| **Reagents** | **1 PCR reaction** | **110 PCR reaction** |
| --- | --- | --- |
| 2 X Master Mix | 12.5 µl | 1375 µl |
| MDR1-SF3 (10 µM) | 0.5 µl | 55 µl |
| MDR1-SR3 (10 µM) | 0.5 µl | 55 µl |
| Nuclease-free water | 9.5 µl | 1045 µl |
| Total | 23 µl | 1. µl |

Add 2 µl PCR product from the primary PCR (region 2-3) to the corresponding tube or well.

PCR cycling condition:

| **One cycle** |  |
| --- | --- |
| 95°C | 5 minutes |
| **25 PCR cycles:** |  |
| 95°C | 30 seconds |
| **56**°C | 45 seconds |
| 72°C | 45 seconds |
| **One cycle** |  |
| 72°C | 10 minutes |
| 4°C | ∞ |
|  |  |

**Preparation of *Pfk13* primary PCR master mix:**

| **Reagents** | **1 PCR reaction** | **110 PCR reaction** |
| --- | --- | --- |
| Nuclease-free water | 9 µl | 990 uL |
| 2X Phusion Master Mix | 12.5 µl | 1375 uL |
| K13_PF1 primer (10 µM) | 1.25 µl | 137.5 ul |
| K13_PR1 primer (10 µM) | 1.25 µl | 137.5 ul |
| Total | 24 µl | 2640 ul |

Add extracted DNA 1 µl into the well.

PCR cycling condition:

| **One cycle** |  |
| --- | --- |
| 98°C | 2 minutes |
| **30 PCR cycles:** |  |
| 98°C | 10 seconds |
| **48**°C | 30 seconds |
| 68°C | 2.3 minute |
| **One cycle** |  |
| 68°C | 10 minutes |
| 4°C | ∞ |

Secondary PCR master mix for a 25-ul reaction:

| **Reagents** | **1 PCR reaction** | **110 PCR reaction** |
| --- | --- | --- |
| Nuclease-free water | 9 uL | 990 uL |
| 2X Phusion Master Mix | 12.5 uL | 1375 uL |
| NSF2 primer (10 µM) | 1.25 uL | 137.5 uL |
| NSR2 primer (10 µM) | 1.25 uL | 137.5 uL |
| Total | 24 uL | 2640 uL |

Make a 1:10 dilution of the primary PCR product by adding 1.0 uL to 9.0 µl nuclease free water.

Add 1.0 µl of the diluted primary PCR product to each well.

PCR cycling condition:

| **One cycle** |  |
| --- | --- |
| 98°C | 2 minutes |
| **30 PCR cycles:** |  |
| 98°C | 10 seconds |
| **54**°C | 30 seconds |
| 68°C | 1 minute |
| **One cycle** |  |
| 68°C | 10 minutes |
| 4°C | ∞ |
