# Supplementary material for: Temporal trends in molecular markers of drug resistance in Plasmodium falciparum in human blood and profiles of corresponding resistant markers in mosquito oocysts in Asembo, western Kenya
Source: Malar J. 2022 Sep 13;21:265. doi: 10.1186/s12936-022-04284-6 (PMC9472345; doi:10.1186/s12936-022-04284-6)
Supplement: Supplementary file 3 — Additional file 3: Table S3. Comparison in prevalence of drug resistance SNPs of parasites between mosquitoes and human blood samples collected by three categories of distances. [file 12936_2022_4284_MOESM3_ESM.docx]

**Additional file 3: Table 3. Comparison in prevalence of drug resistance SNPs of parasites between mosquitoes**

**and human blood samples collected by three categories of distances**

|  |  | **Mosquitoes** | **Human blood <7km** | | **Human blood <14km** | | **Human blood <27km** | |
| --- | --- | --- | --- | --- | --- | --- | --- | --- |
| **Gene** | **SNP** | **Prevalence** | **Prevalence** | **P value** | **Prevalence** | **P value** | **Prevalence** | **P value** |
| ***Pfdhfr*** |  | n=62 | n=75 |  | n=153 |  | n=212 |  |
|  | N51I | 98.4 | 100 | 0.453 | 100 | 0.288 | 100 | 0.226 |
|  | C59R | 95.2 | 96.0 | 1.000 | 97.4 | 0.676 | 97.2 | 0.689 |
|  | S108N | 98.4 | 100 | 0.463 | 100 | 0.288 | 100 | 0.226 |
|  | I164L | 0 | 1.3 | 1.000 | 1.3 | 0.588 | 0.9 | 1.000 |
| ***Pfdhps*** |  | n=58 | n=75 |  | n=151 |  | n=210 |  |
|  | S436H | 1.7 | 10.7 | **0.077** | 11.3 | **0.050** | 10.5 | **0.059** |
|  | A437G | 98.3 | 100 | 0.436 | 100 | 0.278 | 100 | 0.216 |
|  | K540E | 98.3 | 98.7 | 1.000 | 99.3 | 1.000 | 99.5 | 0.387 |
|  | A581G | 1.7 | 1.3 | 1.000 | 0.7 | 1.000 | 1.0 | 1.000 |
| ***Pfcrt*** |  | n=48 | n=74 |  | n=151 |  | n=208 |  |
|  | M74I | 27.1 | 17.9 | 0.108 | 15.2 | **0.084** | 17.3 | 0.152 |
|  | N75D/E | 31.3 | 16.2 | **0.073** | 16.6 | **0.038** | 18.3 | **0.051** |
|  | K76T | 31.3 | 16.2 | **0.073** | 16.6 | **0.038** | 18.3 | **0.051** |
| ***Pfmdr1*** |  | n=65 | n=73 |  | n=151 |  | n=207 |  |
|  | N86Y | 9.2 | 12.3 | 0.596 | 12.6 | 0.503 | 10.1 | 1.000 |
|  | Y184F | 43.1 | 48.0 | 0.610 | 51.0 | 0.302 | 52.2 | 0.255 |
|  | D1246Y | 10.0 | 9.6 | 1.000 | 11.9 | 0.813 | 13.5 | 0.520 |

Note: Initial selection of 7 km distance was based on the upper limit of basic mosquito reproductive unit [41]. Comparison in prevalence of drug resistance SNPs between human blood samples collected by distance and mosquito was conducted using Pearson Chi-square tests (exact version). Results showed the similar trends in prevalence of individual drug resistance SNPs between two hosts regardless of distance in blood sample collection, i.e. the lower prevalence of *Pfdhps*-436H while the higher prevalence of *Pfcrt*-75D/E and *Pfcrt-*76T in mosquitoes than in human blood samples. The P values by distance for the three individual SNPs were similar respectively. The numbers in bold: P<0.05 (significant difference) or 0.05≤P<0.10 (marginal difference).
